# Supplementary material for: The interactome of intact mitochondria by cross-linking mass spectrometry provides evidence for coexisting respiratory supercomplexes
Source: Mol Cell Proteomics. 2017 Dec 8;17(2):216–32. doi: 10.1074/mcp.RA117.000470 (PMC5795388; doi:10.1074/mcp.RA117.000470)
Supplement: Supplemental Data [file supp_RA117.000470_133922_0_supp_23978_fzffwf.zip › spectra_annotation/mito_spectra_annotation/442-1-12-1-5-1.pdf]

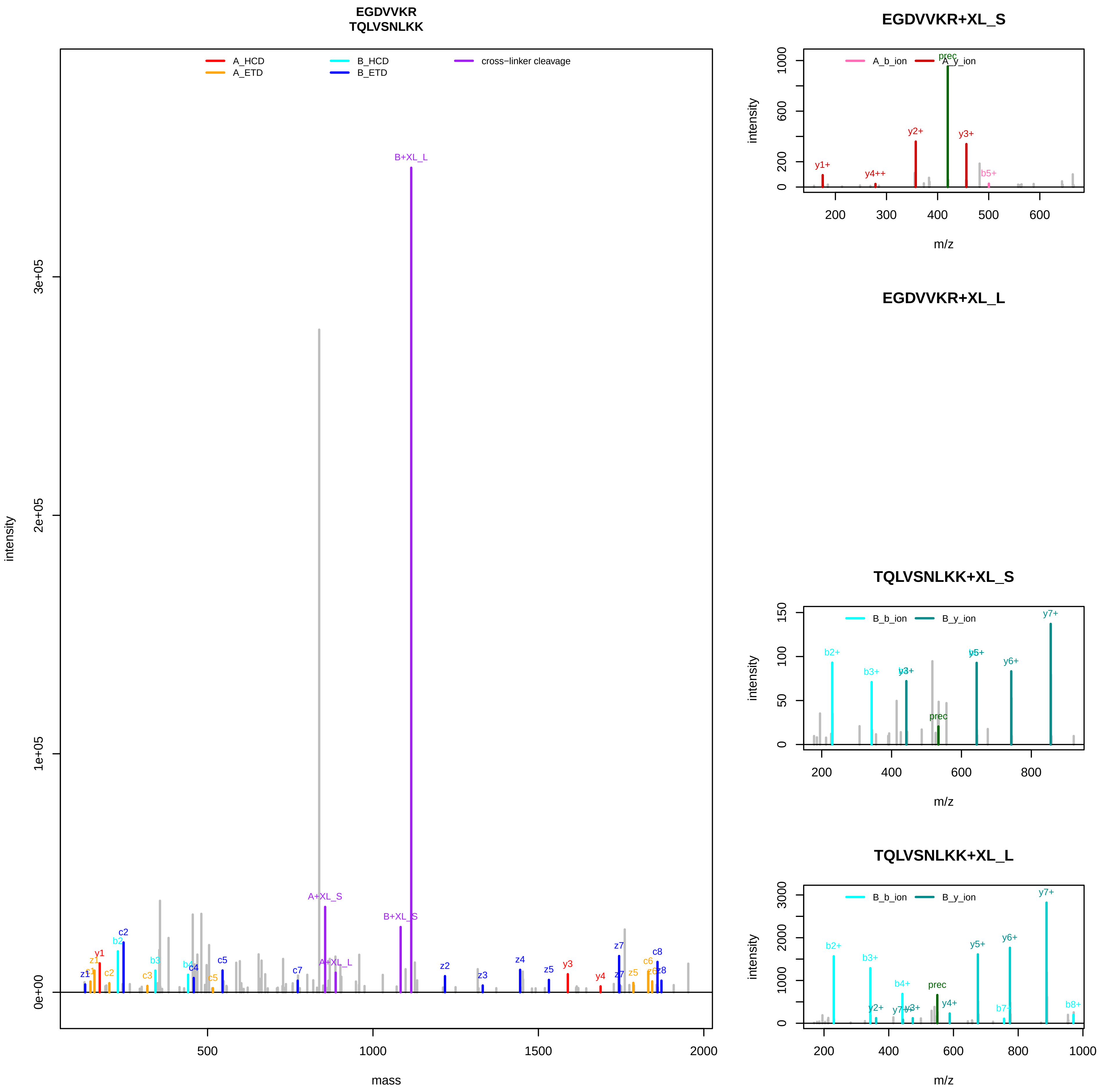
EGDVVKR
TQLVSNLKK
A_HCD
A_ETD
B_HCD
B_ETD
cross−linker cleavage
B+XL_L
A+XL_S
B+XL_S
A+XL_L
intensity
mass
0e+00
1e+05
2e+05
3e+05
500
1000
1500
2000
z1
c1
z1
y1
c2
b2
c2
b3
c3
b4
c4
c5
c5
c7
z2
z3
z4
z5
y3
y4
z7
z7
z5
c6
z6
z8
c8
EGDVVKR+XL_S
A_b_ion
A_y_ion
prec
y1+
y4++
y2+
y3+
b5+
m/z
EGDVVKR+XL_L
TQLVSNLKK+XL_S
B_b_ion
B_y_ion
b2+
b3+
y3+
y5+
y6+
y7+
TQLVSNLKK+XL_L
b4+
y2+
y7++
y3+
y4+
b7+
b8+
